# Cathepsin K Regulates Intraocular Pressure by Modulating Extracellular Matrix Remodeling and Actin-Bundling in the Trabecular Meshwork Outflow Pathway

**DOI:** 10.3390/cells10112864

**Published:** 2021-10-24

**Authors:** Avinash Soundararajan, Sachin Anil Ghag, Sai Supriya Vuda, Ting Wang, Padmanabhan Paranji Pattabiraman

**Affiliations:** 1 Glick Eye Institute, Department of Ophthalmology, Indiana University School of Medicine, 1160 West Michigan Street, Indianapolis, IN 46202-5209, USA; avisound@iu.edu (A.S.); ghags@iu.edu (S.A.G.); svuda@iu.edu (S.S.V.); twa3@iu.edu (T.W.); 2 Stark Neuroscience Research Institute, 320 West 15th Street, Indianapolis, IN 46202-2266, USA

**Keywords:** cathepsin K, trabecular meshwork, intraocular pressure, extracellular matrix, actin cytoskeleton

## Abstract

The homeostasis of extracellular matrix (ECM) and actin dynamics in the trabecular meshwork (TM) outflow pathway plays a critical role in intraocular pressure (IOP) regulation. We studied the role of cathepsin K (CTSK), a lysosomal cysteine protease and a potent collagenase, on ECM modulation and actin cytoskeleton rearrangements in the TM outflow pathway and the regulation of IOP. Initially, we found that CTSK was negatively regulated by pathological stressors known to elevate IOP. Further, inactivating CTSK using balicatib, a pharmacological cell-permeable inhibitor of CTSK, resulted in IOP elevation due to increased levels and excessive deposition of ECM-like collagen-1A in the TM outflow pathway. The loss of CTSK activity resulted in actin-bundling via fascin and vinculin reorganization and by inhibiting actin depolymerization via phospho-cofilin. Contrarily, constitutive expression of CTSK decreased ECM and increased actin depolymerization by decreasing phospho-cofilin, negatively regulated the availability of active TGFβ2, and reduced the levels of alpha-smooth muscle actin (αSMA), indicating an antifibrotic action of CTSK. In conclusion, these observations, for the first time, demonstrate the significance of CTSK in IOP regulation by maintaining the ECM homeostasis and actin cytoskeleton-mediated contractile properties of the TM outflow pathway.

## 1. Introduction

Glaucoma is a major public health concern and the leading cause of irreversible blindness across the globe, afflicting mainly the aging population [1]. About 80 million people have glaucoma worldwide, and it is expected to cross 111.8 million by 2040. The most common type of glaucoma is the primary open-angle glaucoma (POAG), with over 57.5 million people affected in the world [2]. The major risk factor for the development of POAG is elevated intraocular pressure (IOP) [3]. This is the only modifiable risk factor in halting the progression of the disease, which is treated either by surgery or using eye drops [4]. The IOP is generated by the resistance offered to the aqueous humor (AH) drainage via the conventional outflow pathway, comprising the trabecular meshwork (TM), juxtacanalicular tissue (JCT), and the Schlemm’s canal (SC) [5]. The conventional outflow pathway is responsible for nearly 60–80% of AH drainage [6]. The TM tissues are made up of porous beams of endothelial-like cells encapsulated in a collagen matrix [7]. Increased resistance to AH outflow and improper drainage of AH results in ocular hypertension causing optic nerve damage and blindness [4]. Reducing the AH outflow resistance improves the AH outflow drainage and lowers IOP. A 20% reduction in IOP significantly alleviates the risk of developing glaucoma [8,9]. An optimal balance in ECM remodeling in the JCT-TM region is needed for the generation of AH resistance, but excess ECM deposition results in increased resistance and elevated IOP [10]. In POAG, characteristic changes occur in the tissue structure of the AH outflow pathway [11,12,13]. Major changes in glaucomatous eyes are excessive alterations in ECM organization in the JCT-TM region and the accumulation of sheath-like plaque materials, leading to altered stiffness [13,14,15,16,17]. Extensive studies in rodent models demonstrate the importance of ECM homeostasis in the AH outflow pathway. Mutations in genes encoding elastic microfiber components and certain collagen genes are associated with glaucoma [18]. Transgenic mice with a targeted mutation in the collagen 1 gene demonstrate elevated IOP due to improper degradation of fibrillar collagen and increased deposition in the outflow pathway [19,20,21,22]. Contrarily, the inhibition of ECM modifications, such as GAG sulfation, perfusion with GAG-degrading enzymes, and ECM hydrolyzing enzymes, such as matrix metalloproteinases (MMPs), decrease resistance to AH outflow and lowers IOP [23,24,25]. Several studies show altered MMP concentrations in AH from glaucomatous eyes compared to normal [26,27]. As demonstrated by various studies previously, the role of actin cytoskeleton-mediated TM contraction and ECM-mediated TM stiffness play important roles in regulating the AH drainage and IOP [28,29,30]. An increase in actin polymerization leads to increased cytoskeletal tension generated via actomyosin contraction. This tension can be balanced by the accumulation of ECM, causing an increase in matrix-associated stiffness. This interplay between excessive actomyosin contractility and ECM stiffness results in increased AH outflow resistance ensuing in elevated IOP [10,29,31]. On the contrary, pharmacological agents that inhibit the fibrogenic activation, as well as decrease actin polymerization from lowering the actin-based cellular relaxation, aids in lowering the IOP [13,28,32].

Cathepsins are lysosomal proteases with various catalytic types, which comprise serine, aspartyl, and cysteine protease [33]. Cysteine cathepsins, including cathepsin B (CTSB) and cathepsin K (CTSK), are secreted and play an important role in ECM degradation [34]. Multiple studies conducted in the Liton lab at Duke University have shown the lysosomal presence and the significance of CTSB in the intracellular degradation of ECM [35,36,37,38]. Additionally, CTSB is also localized in the caveolae, acting as a local site for protein degradation. Interestingly, CTSB is involved in the regulation of profibrogenic modulators, including plasminogen activator inhibitor (PAI-1) and transforming growth factor β2 (TGFβ2), thereby regulating ECM remodeling and IOP homeostasis [35,36,37]. However, very little information about the expression, distribution, regulation in TM tissue, and the role of CTSK in IOP regulation is available. One report showed that CTSK mRNA was induced under oxidative stress in porcine TM (PTM) cells but not the protein [38]. CTSK is highly effective in the degradation of collagen I, and it requires CTSK to be bound to collagen fibers through glycosaminoglycans (GAGs) [39,40,41]. CTSK is unique among mammalian proteases, as its collagenolytic activity does not depend on the destabilization of collagen triple helix but cleaves collagen at various sites in contrast to other cysteine proteases [42]. CTSK also effectively degrades elastin fibers [43]. Cysteine proteases are activated and functional at a low pH, and most of them are unstable and inactive at a neutral pH [44]. CTSK is an exception, as it is activated at a lower pH or by GAGs, such as chondroitin sulfate, and remains functional in the pH range of 5.0–7.4 [40,45,46,47]. Therefore, CTSK is functional after it is secreted into the extracellular milieu. The secretion of CTSK is a regulated process, and laminar shear stress can induce CTSK secretion, as seen in mouse aortic endothelial cells [48]. Beyond its own ECM degrading function, CTSK can activate matrix metalloprotease 9 (MMP9), which is involved in ECM degradation [49]. In bone and lung tissues, an aberrant CTSK activity is associated with inadequate collagen turnover [50,51,52]. The absence of CTSK is reported to alter actin structures in osteoclasts [53,54]. Interestingly, in CTSK deficient mice, the levels of transforming growth factor β1 (TGFβ1) are increased due to the loss of proteolytic control of CTSK on TGFβ1 [55]. On the contrary, in the zebrafish model of chondrogenesis, there is a positive relationship between CTSK activation and TGFβ signaling [56,57]. The TGFβ2 protein levels are elevated in glaucomatous TM, optic nerve head, and AH [58,59,60]. These increased levels of TGFβ2 are associated with increasing ECM, as well as IOP [60,61,62]. Intriguingly, it has been reported that mRNA levels of CTSK are increased in the glaucomatous human lamina cribrosa cells [63].

With very little understanding of the regulation and role of CTSK in the TM AH outflow biology, this study explores the mechanistic role of CTSK on IOP by regulating ECM remodeling, cytoskeletal dynamics, and modulation of TGFβ2 availability in the TM.

## 2. Materials and Methods

### 2.1. Materials

Human recombinant CTGF (R&D systems, Minneapolis, MN, USA, #9190CC), Human recombinant TGFβ2 (MilliporeSigma, St. Louis, MO, USA, #GF440), human recombinant endothelin1 (MilliporeSigma, #E7764-50UG), dexamethasone (MilliporeSigma, #265005-100MG), and Balicatib (Tocris, Minneapolis, MN, USA, #5585) were used in this study. Primary antibodies used for the study are Rabbit anti-fibronectin (gift from Dr. Harold Erickson, Duke University), anti-collagen1α (Abcam, Cambridge, MA, USA, #ab34710), anti-elastin (Proteintech Group Inc., Rosemont, IL, USA, #15257-I-AP), anti-TGFβ2 (Abcam, Cambridge, MA, USA, #ab36495), mouse anti-cathepsin K (Santa Cruz Biotechnology, Dallas, TX, USA, #sc-48353), anti-α-smooth muscle actin (αSMA) (MilliporeSigma, #A2547), anti-TGFβ2 (Abcam #ab36495), anti-Vinculin (MilliporeSigma, #V9131), anti-Fascin-1 (Santa Cruz Biotechnology, #sc-46675) anti-total cofilin (Cell Signaling Technology, Danvers, MA, USA, #5175S), anti-phoshpo cofilin (Cell Signaling Technology, Danvers, MA, USA #3311S), anti-lysosome-associated membrane protein-2 (LAMP2) (Invitrogen, Waltham, MA, USA, #PA1-655) with anti-GAPDH (Santa Cruz Biotechnology, #sc-25778), anti-β actin (MilliporeSigma, #A1798), and anti-β tubulin (Invitrogen, #PA1-16947) as loading controls. Secondary antibodies, conjugated horseradish peroxidase, used are Donkey anti-mouse IgG (#715-035-150) and Donkey anti-rabbit IgG (#715-035-144), which were purchased from Jackson Immuno Research, West Grove, USA. Secondary antibodies, conjugated fluorescence, used are Goat anti-Mouse IgG Alexa Fluor Plus 488 (#A32723), Goat anti-Mouse IgG Alexa Fluor Plus 594 (#A32742), Goat anti-Rabbit IgG Alexa Fluor Plus 488 (#A32731), and Goat anti-Rabbit IgG Alexa Fluor Plus 594 (#A32740), which were purchased from Invitrogen. Green fluorescent-labeled Phalloidin (Alexa Fluor Plus 488 PHA, #A12379) was purchased from Invitrogen for filamentous actin (F-actin) staining. SiR-actin Kit (Cytoskeleton Inc., Denver, USA, #CY-SC001) containing SiR-actin and verapamil was used for performing live cell-imaging. Fluorescein Diacetate (FDA) (#F7378-5G) and Propidium Iodide (PI) (#P4170-25MG) was purchased from Millipore Sigma.

### 2.2. Primary Trabecular Meshwork Cell Culture

Primary human TM cells were cultured from TM tissue isolated from the leftover donor corneal rings after they had been used for corneal transplantation at the Indiana University Clinical Service, Indianapolis, as described previously [64]. HIPPA compliance guidelines were adhered to for the use of human tissues. The usage of donor tissues was exempt from the DHHS regulation and the IRB protocol (1911117637), approved by the Indiana University School of Medicine IRB review board. The age, race, and sex of the donors were obtained from eye banks, which provided the corneas. TM tissue extracted from the corneal ring was chopped into fine pieces and placed in a 2% gelatin-coated 6-well tissue culture plate sandwiched by a coverslip. The tissues were grown in OptiMEM (Gibco, #31985-070), containing 20% FBS and penicillin-streptomycin-glutamine solution (Gibco, #10378-016). The expanded population of HTM cells was subcultured after 1–2 weeks in DMEM, containing 10% FBS and characterized by detection of dexamethasone-induced myocilin [64].

Primary porcine TM (PTM) cells were cultured from fresh porcine TM globes obtained from Indiana Packers, Delphi, IN, USA, which were transported on ice within 2 h of sacrifice. The isolated TM tissue was finely chopped and enzymatically dissociated, using a solution containing 10 mg collagenase type 4 (Worthington Biochemical, Lakewood, CL, USA, #9007-34-5), and 3 mg human serum albumin (MilliporeSigma, #A4329) in 199 media (Gibco-ThermoFisher, Waltham, MA, USA, #11150-059) at 37 °C for 1 h in a shaker. The cells were pelleted by centrifugation at 2500 rpm for 12 min, collected, and plated in a 2% gelatin-coated plastic tissue culture plate with DMEM containing 20% FBS and penicillin-streptomycin-glutamine solution. The expanded population of PTM cells was sub-cultured in DMEM containing 10% FBS. The PTM cells was maintained at 37 °C under 5% CO_2_. All experiments were conducted using confluent HTM or PTM cultures as mentioned, using cells in between passage four to six, and were performed after overnight serum starvation unless mentioned, otherwise.

All experiments in this manuscript were performed using biological replicates.

### 2.3. Replication-Deficient Recombinant Adenovirus Expressing CTSK

Replication-defective recombinant adenovirus expressing secretory CTSK (AdCTSK) (VH822027) with CMV promoter-driving CTSK expression with a C-terminal FLAG and His tags in pAD vector was purchased from Vigene Biosciences Inc, Rockville, MD, USA. A control empty virus (AdCTL) was a gift from Dr. Douglas Rhee, Case Western Reserve University, Cleveland, OH, USA. An Adeno-X™ Rapid Titer Kit (Takara Bio Inc, San Jose, CA, USA, #632250) was used to determine the presence and optimum viral load for infection. HTM cells were infected with either AdCTL or AdCTSK with 50 multiplicities of infection. After 72 h of treatment, the cells were washed with 1X PBS and collected using either TRIZOL for extracting RNA or 1X RIPA for protein extraction. Media was collected and concentrated using Nanosep^®^ Centrifugal Devices with Omega™ Membrane 10K (Pall, Port Washington, WI, USA, #OD010C34).

### 2.4. Cyclic-Mechanical Stretch Experiment

HTM cells were cultured in collagen-coated plates (Flexcell International, Burlington, VT, USA, BioFlex plates, BF-3001C), and after 90% confluency, the plates were fixed on a FlexCell (Flexcell International, FX-6000 Tension System). The cells were stretched at 0.69 Hz frequency with 15% stretching for 8 h for gene expression analysis and 24 h for protein expression analysis based on previously published reports [65,66,67,68]. Cells and media were collected and processed, as mentioned later, for RNA analysis and immunoblotting.

### 2.5. Ex Vivo Elevated IOP Model

Freshly enucleated porcine whole globes obtained were cleaned by trimming the extra-ocular muscle and immersing in povidone-iodine topical antiseptic solution (Betadine), followed by washes in 1x PBS solution. The globes were then cut below the limbus, and anterior segments with intact TM were prepared by removing the remnant of the vitreous humor, lens, ciliary body, and iris. The anterior segments were then mounted onto a custom-made perfusion chamber consisting of a foundational component and an O-ring. The foundational component consists of a convex mound with two cannulas that allow for an influx of fluid and its subsequent drainage to simulate the AH pathway. The column connected to the chamber was filled with 1X PBS containing 5.5 mM D-glucose, up to a height that corresponds to either 15 or 30 mmHg. A baseline pressure of 15 mmHg was first established for 30 min for all the eyes. One eye was subjected to 15 mmHg pressure, whereas the contralateral eye was subjected to a constant pressure of 30 mmHg for 5 h. Then TM was collected and processed for either RNA or protein as described below.

### 2.6. Ex Vivo Inhibition of CTSK Activity in Porcine Anterior Segment Perfusion Culture and Measurement of IOP

After preparing the porcine anterior segments as mentioned above, the perfusion chamber containing the eye was connected to a perfusion pump (inflow) and transducer (outflow) using tubing. Transducers were connected to a bridge amplifier, which is in turn connected to a power lab, which sends signals to a computer where the IOP is recorded using Lab Chart software (LabChart; ADInstruments, Colorado Springs, CO, USA). A baseline pressure was established for nearly 24 h by perfusing serum-free DMEM media with 10% antibiotic-antimycotic solution (Corning, #15240-062) into the anterior segment using a 10 mL syringe at a constant rate of 3 µL/min mounted to a high precision perfusion pump (Fusion 200-X, Chemyx Inc., Stafford, USA). After achieving a stable baseline, two study groups were established: control eyes that received a sham DMSO treatment and the experimental eyes that received 10 µM balicatib. IOPs were recorded every second and averaged per hour. Eyes that experienced contamination showed erroneous IOP recordings in the negative pressure range or readings above 50 mmHg during the first 24 h were considered a failure. Such negative IOP recordings were observed, for instance, when debris blocked the transducer lumen while early high IOP was interpreted as relative TM failure or blockage. The changes in IOP (ΔIOP) were calculated as the change in pressure relative to the baseline of drug/sham perfusion. At the end of the perfusion studies, the TM tissues were either sectioned for histology studies and fixed with 4% paraformaldehyde or the TM was collected for protein analysis.

### 2.7. Cell Viability Assay

The cell viability assay was performed using FDA and PI staining. PTM cells were grown in 6-well plates until they attained 90% confluency. The cells were treated with a final concentration of 10 µM balicatib for 24 h followed by overnight serum starvation. FDA-PI double staining was performed based on a previously published protocol with minor modifications [69]. Briefly, after subsequent treatment, the cells were washed with 1X PBS twice after aspirating the culture medium. Then FDA-PI working solution was added with final concentrations of FDA: 10 μg/mL, and PI: 100 μg/mL, in 1X PBS and incubated at 37 °C for 5 min in the dark. After incubation, the FDA-PI working solution was aspirated and washed once with chilled 1X PBS. The cells were imaged using a Zeiss fluorescence microscope for the fluorescence emissions of FDA and PI at 520 and 620 nm, respectively, and under phase contrast mode for light microscopy. While imaging, chilled 1X PBS (100 μL/well) was added to prevent the cells from drying.

### 2.8. CTSK Activity Assay

CTSK activity under various treatments was assessed using a CTSK activity assay kit (BioVision Inc., Milpitas, USA, #K141-100) and the suggested protocol with minor modifications [70]. Cells were lysed using chilled CK cell lysis buffer, followed by incubation on ice for 10 min and centrifuged at top speed for 5 min to collect the supernatant for the experiment. Along with cell lysate, conditioned media was also collected and concentrated using 10K cut-off centrifugal devices and collected using a hypotonic solution consisting of 10 mM Tris buffer and 0.2 mM MgCl_2_. To ensure an equal loading, protein concentration was estimated using the Bradford method (Bio-Rad, Hercules, USA, #5000006), a 50 µL sample was loaded into 96 well solid white flat bottom microplate, and 50 µL of CK Reaction buffer was added to each sample. It was then followed by the addition of 2 µL of 10 mM CK substrate Ac-LR-AFC to a final concentration of 200 µM. For the negative control, 2 µL of CK inhibitor was used. The samples are then incubated at 37 °C for 2 h. Post incubation samples were read at 400 nm excitation and 505 nm emission. Fold-increase in CTSK activity was determined by comparing the relative fluorescence units with the level of uninduced control or negative control sample.

### 2.9. Gene Expression Analysis

Total RNA was extracted from TM tissue and cells using the Trizol method following the manufacturer’s protocol. The RNA amounts were quantified using NanoDrop 2000 UV-Vis Spectrophotometer (Thermo Scientific, Wilmington, DE). Equal amounts of RNA were then reverse transcribed to cDNA using the 5X All-In-One RT MasterMix (Applied Biological Materials Inc, Richmond, Canada, #G492) with genomic DNA removal according to the manufacturer’s instructions. The following reaction condition was maintained for cDNA conversion: incubation at 25 °C for 10 min, followed by incubation at 42 °C for 15 min, and enzyme inactivation at 85 °C for 5 min. The cDNA was diluted as per requirement, and 10 µL was used for gene expression analysis using Quant Studio Flex 6/7 thermocycler (ThermoFisher Scientific, Waltham, USA). Bright Green 2X qPCR MasterMix-ROX (Applied Biological Materials Inc., MasterMix-LR-XL) and gene-specific oligonucleotides (Integrated DNA Technologies, Coralville, USA) were used for the analysis. Sequence-specific forward and reverse oligonucleotide primers for the indicated genes are provided in Table 1. Each sample for the PCR reaction was performed in triplicate using the following protocol: initial denaturation for 95 °C for 2 min followed by 40 cycles of denaturation at 95 °C for 15 s, annealing at 60 °C for 15 s, and extension at 72 °C for 1 min. An extended step was used to measure the melting curves obtained immediately after amplification by increasing the temperature in 0.4 °C increments from 65 °C for 85 cycles of 10 s each. The fold difference in expression of test genes between the control and treatment was calculated by the delta-delta Ct method [71]. Normalization was performed using either (hydroxymethylbilane synthase (HMBS) for PTM or glyceraldehyde-3-phosphate dehydrogenase (GAPDH) for HTM.

### 2.10. Protein Analysis by Immunoblotting

The cell lysates containing total protein were prepared using 1X RIPA buffer composed of 50 mM Tris-HCl (pH 7.2), 150 mM NaCl, 1% NP-40, 0.1% SDS, 1 mM EDTA, and 1 mM PMSF with protease and phosphatase inhibitors (ThermoFisher Scientific, #A32961) and then sonicated. The protein concentration was determined using either the Bradford Assay Reagent/Bio-Rad Protein Assay Kit I (Bio-Rad, 5000001) or BCA protein assay (ThermoFisher Scientific, #23338). A total of 20–100 μg of the protein sample was mixed with 4X Laemmli buffer and separated on 8–15% SDS polyacrylamide gel. Following the gel run, the proteins were transferred to 0.45 µM pore size nitrocellulose membrane (GE Healthcare, Chicago, USA, #10600003). Ponceau S staining of the membrane was performed to document the protein loading after transfer. Membranes were blocked in 5% non-fat dry milk in Tris-buffered saline with 0.1% Tween for 2 h followed by respective primary antibodies overnight at 4 °C (~16 h), and then by horseradish peroxidase-conjugated secondary antibodies (Jackson Immuno Research). The blots were washed with 1X TBST, and the immunoreactivity was detected using Western Lightning Plus Enhanced Chemiluminescence (ECL) Substrate (Perkin Elmer, Shelton, USA) and imaged in a ChemiDoc MP imaging system (Bio-Rad). Blots were stripped using mild stripping buffer if required to reprobe for the loading control and multiple proteins within the same molecular weight range. The data were normalized to GAPDH or β-tubulin. Semi-quantitative analyses and fold changes were calculated from the band intensities measured using Image J software.

### 2.11. Protein Distribution Analysis by Immunohistochemistry and Immunofluorescence

Tissue sections from formalin-fixed, paraffin-embedded human donor whole globes eye were prepared at Case Western Reserve University, Cleveland, Ohio. Paraffin-embedded porcine TM tissue slides were prepared at the Histology Core, Indiana University, and immunolabeling was performed. Briefly, five-micron thick tissue sections were deparaffinized in xylene for 15 min and incubated with xylene for a second 15 min period. The sections were subsequently hydrated with ethanol dilutions (100%, 95%, and 70%). To unmask the antigen epitopes, heat-induced antigen retrieval was performed using 0.1 M citrate buffer pH 6.0 (Vector Laboratories, Burlingame, CA, USA) for 20 min at 100 °C. The slides were then blocked for nonspecific interactions with Sniper Background Reducer (BS966H) (Biocare Medicals, Pacheco, USA). For immunohistochemical staining, hematoxylin and eosin stainings were performed using Hematoxylin and Eosin Stain kits (Vector Laboratories, Inc.). For immunofluorescence staining, the primary antibody was used at 1:100 dilution overnight at 4 °C. The slides were subsequently washed with 1×PBST, and secondary antibodies were applied at 1:200 for 1 h at RT. After two additional washes, the coverslip was mounted with Fluoroshield mounting medium with DAPI (Abcam, #ab104139). For staining, a minimum of 2 slides per ocular tissue sample were utilized, with each slide containing 3 consecutive sections from the same eye. This was repeated across the biological replicates tested.

HTM cells were grown on a 2% gelatin-coated glass coverslips until they attained 90% confluency. After appropriate treatment, the cells were washed with 1X PBS twice, fixed in 4% paraformaldehyde for 15 min, permeabilized with 0.2% triton-x-100 in PBS buffer for 10 min, and blocked with 5% bovine serum albumin in 1X PBS for 1 h. The cells were then incubated with the respective primary antibody overnight at 4 °C. After washing twice with 1X PBS, they were incubated in Alexa fluor-conjugated secondary antibodies for 2 h at room temperature. Finally, the coverslips were washed and mounted onto glass slides with Fluoroshield Mounting Medium (Abcam, #ab104139).

All the slides were observed under a Zeiss LSM 700 confocal microscope, z-stack images were obtained and processed using Zeiss ZEN image analysis software.

### 2.12. Live-Cell Imaging

Live-cell imaging for observing changes in filamentous actin was performed using a SiR-actin Kit. HTM cells were grown on a 2% gelatin-coated glass bottom multi-well plates (Cellvis, Mountain View, USA, #D35C4-20-1.5-N). When the cells reached the desired density, the cells were first infected with either AdCTL or AdCTSK. After 10 h, actin staining solution containing 1 μM SiR-actin and 10 μM verapamil was added. The cells were maintained in the incubator at 37 °C in a humidified atmosphere containing 5% CO_2_. After 24 h, live-cell imaging was performed at 37 °C in a Tokai Hit Stage Top incubator to record the changes in actin structures under a Zeiss LSM 700 confocal microscope. The z-stack images were obtained and processed using Zeiss ZEN image analysis software.

### 2.13. Quantitative Image Analysis

ImageJ (version 1.53a) software was used to analyze the collagen and fibronectin fluorescence intensity in IF tissue images. Specifically, the immunofluorescence image obtained from the tissue section was converted into an 8-bit image, then the threshold default setup under Adjust was used to convert the image from grayscale into a binary image. Next, the region of interest (ROI) tool was chosen for analysis. In this analysis, different ROIs (equal area) in the TM outflow pathway were chosen in an image, and the intensity was measured and compared between control and balicatib.

### 2.14. Statistical Analysis

All data are presented as the mean ± standard error of the mean (SEM) of a minimum of three to six independent observations. GraphPad Prism 8 was used to generate graphs. Quantitative data were analyzed by the Student’s paired or unpaired *t*-test, and a *p*-value ≤ 0.05 was considered statistically significant

## 3. Results

### 3.1. CTSK Is Expressed in the Human TM Outflow Pathway and Is Regulated by Factors That Modulate IOP

**Figure 1:**
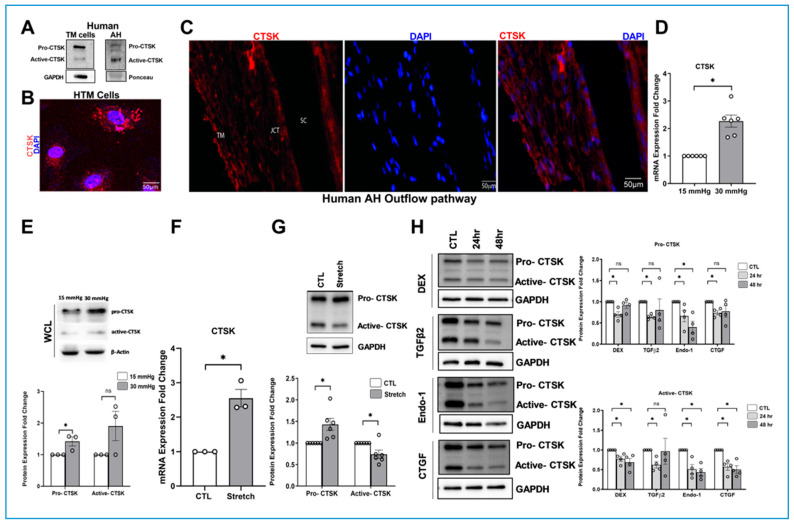
Expression, distribution, and regulation of CTSK in human AH outflow pathway. (**A**) Expression of CTSK in primary HTM cells (donor aged 68y, male, Caucasian) and in AH from a human donor eye (donor aged 74y, female, Caucasian). Pro-CTSK is seen at ~37 kDa, and active-CTSK is seen at ~27 kDa. GAPDH was used as the loading control for cell lysate, and Ponceau S-band at ~55 kDa was used for the AH. (**B**) Immunofluorescence showing the cytosolic distribution of CTSK (red puncta) in primary HTM cells. The nucleus was stained with DAPI in blue. Images were captured in z-stack in a confocal microscope, and stacks were orthogonally projected. Scale bar 50 microns. (**C**) Tissue distribution of CTSK in the AH outflow pathway of a normal human eye specimen by immunofluorescence. The representative image shows the CTSK distribution (red) in the TM-JCT region and in the inner wall of SC. DAPI was stained in blue. Scale bar 50 microns. (**D**–**H**) Regulation of CTSK mRNA and protein expression levels by stressors involved in modulating IOP. (**D**) mRNA and (**E**) pro-CTSK protein expression significantly increased in TM derived from enucleated porcine anterior segments perfused under the elevated pressure of 30 mmHg for 5 h compared to 15 mmHg. No significant change in the active-CTSK protein level was observed. (**F**) mRNA and (**G**) protein expression significantly increased in HTM cells subjected to cyclic mechanical stretch compared to non-stretched control. Active-CTSK protein demonstrated a significant decrease. (**H**) CTSK protein levels in response to 24 and 48 h treatment of steroid dexamethasone (DEX), transforming growth factor β2 (TGFβ2), endothelin-1 (Endo-1), and connective tissue growth factor (CTGF). Pro- and active-CTSK were significantly decreased in the 24 h treatment with DEX, TGFβ2, Endo-1, and CTGF and 48 h Endo-1 treatment. Active-CTSK was significantly decreased in 48 h Dex and CTGF treatments. The results were based on semi-quantitative immunoblotting with subsequent densitometric analysis. HMBS and GAPDH for PTM and HTM, respectively, were used as internal controls for qPCR analysis. β-actin or GAPDH was used as a loading control for immunoblotting analysis. Circles in the histograms represent sample numbers. Values represent the mean ± SEM, where *n* = 3–6 (biological replicates). * *p* ≤ 0.05 was considered significant and n.s. denotes non-significant.

To determine the protein expression and secretion of CTSK in TM and AH, protein extracted from human donor HTM tissue and AH were immunoblotted. We identified two bands of CTSK protein-pro-CTSK (~37 kDa) and active-CTSK (~27 kDa) (Figure 1A), indicating that CTSK is expressed in TM and secreted into the AH. Following the confirmation of CTSK expression, we performed immunofluorescence analysis and confocal imaging to ascertain the distribution of CTSK in HTM cells and in the human TM outflow pathway. The cytosolic presence of CTSK was seen in the form of punctate (red puncta) distribution within the HTM cells (Figure 1B). Furthermore, the CTSK distribution in the anterior chamber angle of a normal human eye was identified by CTSK immunopositive cells in the TM-JCT region, as well as the endothelial cells of the inner wall of SC (Figure 1C).

The enzyme CTSK is involved in ECM degradation [42], and ECM plays an important role in IOP homeostasis [10]. Therefore, we investigated the effects of various stressors, known to increase ECM in the TM outflow pathway and elevate IOP, on CTSK protein levels. Cyclic changes in the IOP induced by ocular pulse can affect TM tissue by subjecting the TM to constant mechanical stress [72]. To evaluate the effects of mechanical stress on TM, we utilized two strategies: (a) elevated IOP stress [73]—where porcine anterior segments were subjected to 2x pressure—and (b) HTM cells were subjected to cyclic mechanical stretch [65,74]. Under elevated pressure stress (2x), we found that CTSK mRNA in TM tissue increased significantly (*n* = 6, *p* = 0.0002) (Figure 1D). This was also reflected in the significant increase in pro-CTSK (*n* = 3, *p* = 0.05) in 30 mmHg compared to 15 mmHg, but the active-CTSK did not show any change (Figure 1E). TM subjected to cyclic mechanical stretch showed an increase in mRNA levels (*n* = 3, *p* = 0.003) (Figure 1F) and the examination of CTSK protein showed a significant increase in pro-CTSK (*n* = 6, *p* = 0.003), whereas the active-CTSK decreased significantly (*n* = 6, *p* = 0.02) (Figure 1G). In addition, we also explored whether CTSK is regulated by dexamethasone (DEX), TGFβ2, Endothelin 1 (Endo-1), and connective tissue growth factor (CTGF); each of which has been implicated in elevated IOP and glaucoma [75,76,77]. With *n* = 4, a time-dependent study with a constant dose treatment of the above-mentioned factors (Figure 1H) resulted in: (a) at 24 h, pro- and active-CTSK levels were significantly decreased—DEX (*p* = 0.003 and 0.01), TGFβ2 (*p* = 0.0001 and 0.003), Endo-1 (*p* = 0.001 and 0.002), and CTGF (*p* = 0.001 and 0.002), and (b) at 48 h, Endo-1 showed a significant decrease in both pro- and active-CTSK (*p* = 0.004 and 0.0009), and only active-CTSK showed a significant decrease in DEX (*p* = 0.01) and CTGF (*p* = 0.002). All experiments mentioned above were performed and analyzed using biological replicates. These results imply that CTSK is tightly regulated under stress, and except 2x pressure stress, all the other stressors known to elevate IOP and participate in glaucoma pathogenesis were able to lower the functional CTSK (active form).

### 3.2. Pharmacological Inhibition of CTSK Elevates IOP by Increasing the COL1A Deposition in TM

**Figure 2:**
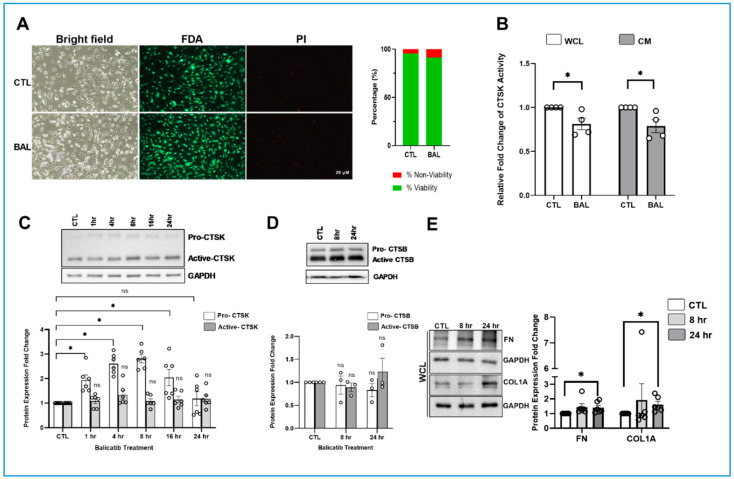
Pharmacological inhibition of CTSK increases ECM expression. (**A**) Examination of the effects of 10 µM balicatib on primary PTM cell viability. Cell viability assay in serum-starved PTM cells treated with 10 µM balicatib was performed using fluorometric live/dead staining of Fluorescein Diacetate/Propidium Iodide (FDA)/(PI) staining. The top panel represents the control DMSO treatment, and the bottom panel represents 10 µM balicatib treatment of serum-starved PTM cells. The first column is a bright field image of PTM cells 24 h after treatment, the second column is FDA staining, and the third column comprises PI staining. The green channel shows viable cells that take up FDA and emit green fluorescence, and the red channel shows dead cells, which takes up PI and gives out red florescence. The graphical representation denotes the mean percentage ratio of FDA/PI, indicating the viable cells (green bar) and the non-viable cells (red bar) in both control and 10 µM balicatib treated cells. (**B**) The relative fold change of CTSK activity from WCL and CM between control and balicatib treatment in PTM cells. A significant reduction in CTSK activity was observed in balicatib-treated compared to the control in WCL and CM. (**C**) The time-dependent effect of balicatib on CTSK protein expression in PTM cells. Balicatib treatment-induced inhibition of CTSK activity demonstrated a significant increase in pro-form of CTSK at 1, 4, 8, and 16 h compared to control, but came back to baseline by 24 h with no significant change in the active-CTSK. (**D**) Analysis of the lysosomotropic activity of balicatib on CTSB. Balicatib treatment did not show any induction of the pro- or active-CTSB levels at 8 or 24 h of treatment, demonstrating little effect on the expression of CTSB in PTM cells. (**E**) Analysis of time-dependent ECM protein expression changes in PTM cells treated with 10 µM balicatib. FN and COL1A showed a significant upregulation at 24 h of balicatib treatment compared to the DMSO control. The results were based on immunoblot analysis with subsequent densitometric analysis. GAPDH was immunoblotted as the loading control. Circles in the histograms represent sample numbers. Values represent mean ± SEM, where *n* = 3–6 (biological replicates). * *p* ≤ 0.05 was considered significant and n.s. denotes non-significant.

Since we identified the negative regulation of functional CTSK by various stressors, we hypothesized that inhibiting CTSK activity can potentially cause an elevated IOP by changing the ECM remodeling in the outflow pathway. Therefore, to understand the effect of loss of CTSK activity on IOP and ECM, we utilized balicatib, a cell-permeable pharmacological inhibitor of CTSK [78] in PTM cells and porcine organ perfusion cultures. Initially, to test if balicatib caused any notable cytotoxicity, we performed a cell viability assay in PTM cells based on live cell labeling for the enzymatic hydrolysis of FDA and nuclear labeling using PI staining. We found that 10 µM balicatib treatment did not show any comparable loss of cell viability compared to the DMSO control (Figure 2A). Since balicatib is a cell-permeable CTSK inhibitor [78,79], we tested for CTSK activity inhibition with and without balicatib treatment in PTM cells. Figure 2B shows a significant reduction in CTSK activity in whole-cell lysate (WCL) (*n* = 4, *p* = 0.03) and in conditioned media (CM) (*n* = 4, *p* = 0.03) in balicatib treatment compared to control. Further, to determine if this lowering of CTSK activity resulted in any changes in CTSK protein expression, we performed a time-dependent study to assay for pro- and active-CTSK protein in whole-cell lysate. We found that the protein expression of pro-CTSK significantly increased at 1 h (*n* = 6, *p* = 0.001), 4 h (*n* = 6, *p* = 0.0001), 8 h (*n* = 6, *p* = 0.0001), and 16 h (*n* = 6, *p* = 0.01) of balicatib treatment in PTM cells compared to the control but came back to baseline by 24 h (Figure 2C). There were no changes in the protein levels of the active-CTSK within the time points tested. Thus, indicating that balicatib can decrease the activity of CTSK and potentially block the functionality of CTSK. Balicatib has been shown to have off-target effects on levels of other cathepsins, such as cathepsin B (CTSB), as it is a lysosomotropic agent [80,81]. Parallelly, CTSB has been studied in a TM outflow pathway [35,36,37] and can behave similar to CTSK, as it is a collagenase and is secreted [36]. Hence, it was important to show if balicatib was upregulating CTSB protein expression as a compensatory mechanism of inhibiting CTSK activity. Therefore, we analyzed the protein expression of CTSB in PTM cells treated with 10 µM balicatib for 24 h compared to the DMSO control. We found no significant changes in the levels of pro- and active-CTSB between treatments, indicating that balicatib did not change CTSB protein levels (Figure 2D). As we have established that balicatib inhibits CTSK activity, we further went on to evaluate the role of CTSK in regulating ECM expression. In PTM cells in vitro, we studied the effects of balicatib after treatment for 8 and 24 h on the changes in the ECM protein levels in WCL. We found a significant increase in ECM proteins, including FN (*n* = 6, *p* = 0.02) and COL1A (*n* = 6, *p* = 0.01), at 24 h post balicatib treatment (Figure 2E). These results show that a loss of CTSK activity can increase ECM levels.

**Figure 3:**
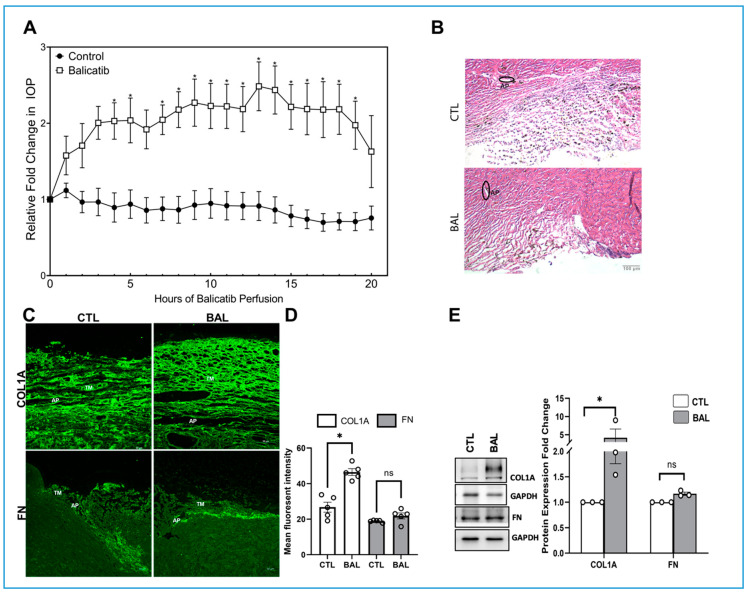
Effect of balicatib on IOP in enucleated porcine eyes. Freshly enucleated porcine eyes were perfused with 10 μM balicatib or DMSO control after baseline was established with perfusion media containing D-glucose at 37 °C. (**A**) Graphical representation of relative fold change in IOP showed a significant increase post 4 h of balicatib perfusion except for the 6 h and remained significant until 19 h compared to vehicle DMSO perfused control. (**B**) Histological examination of the outflow pathway tissues using Hematoxylin and Eosin (H&E) staining showed no gross morphological alterations between balicatib perfusion (bottom panel) compared to the control (top panel). (**C**) Immunolocalization of ECM proteins, including COL1A and FN (green), in the outflow pathway tissue in DMSO control or balicatib. In the balicatib perfused eyes, COL1A (top panel) showed an increased distribution in the TM-JCT region, whereas FN (bottom panel) distribution did not change remarkably compared to the vehicle DMSO perfused tissue. AP indicates aqueous plexus. TM indicates trabecular meshwork. Images were captured in z-stack in a confocal microscope, and stacks were orthogonally projected. (**D**) Quantification of immunofluorescence images using ImageJ-based fluorescence intensity measurements showed a significant increase in COL1A’s mean fluorescence intensity in the TM outflow pathway. (**E**) Quantitative analysis of protein expression changes in COL1A and FN in TM from 10 μM balicatib perfused TM tissues showed a significant increase in COL1A with no change in FN levels compared to vehicle DMSO perfused control. The results were based on immunoblot analysis with subsequent densitometric analysis. GAPDH was immunoblotted as the loading control. Circles in the histograms represent sample numbers. Values represent mean ± SEM, where *n* = 3–5 (biological replicates). * *p* ≤ 0.05 was considered significant and n.s. denotes non-significant.

To understand the role of CTSK in IOP regulation, freshly enucleated porcine eyes were perfused with either 10 μM balicatib or vehicle DMSO for 24 h after the establishment of a baseline for up to 20 h. The IOP readings were monitored every hour up to 24 h. Following the perfusion of 10 μM balicatib, there was a significant increase in IOP from 4 h (*n* = 5, *p* = 0.03) and the significant increase (*n* = 5, *p* ≤ 0.05) sustained from 7 to 19 h post balicatib perfusion (Figure 3A). The increase in IOP in balicatib perfused eyes signified that the inhibition of CTSK activity can alter IOP. To determine the effects of balicatib perfusion on the TM outflow pathway, we analyzed the histological and immunohistochemical changes in the TM outflow pathway. At the outset, the H and E staining did not show any marked difference in the AH outflow regions (Figure 3B). Immunofluorescence images of the ECM proteins, including COL1A and FN (green staining), captured in z-stack on a confocal microscope showed a marked increase in COL1A immunostaining in the TM-JCT region compared to the control (Figure 3C). ImageJ-based quantification of fluorescence intensity in the immunofluorescence image showed a significant increase in COL1A staining (*n* = 5, *p* = 0.0004) in the TM outflow pathway (Figure 3D). On the other hand, the distribution of FN in the TM outflow pathway did not show much change (Figure 3C,D) due to CTSK inhibition by balicatib perfusion. To quantify such changes, we performed immunoblotting with the protein extracted from the TM tissue from balicatib and DMSO perfused eyes. In agreement with immunofluorescence, we found that COL1A showed a significant increase (*n* = 3, *p* = 0.003) and the FN levels were not significant (Figure 3E). This data demonstrates that the inhibition of CTSK activity or loss of CTSK function can significantly increase ECM in the TM outflow pathway and elevate IOP.

### 3.3. CTSK Inhibition Increases Actin Cytoskeleton Bundling and Focal Adhesion Redistribution

**Figure 4:**
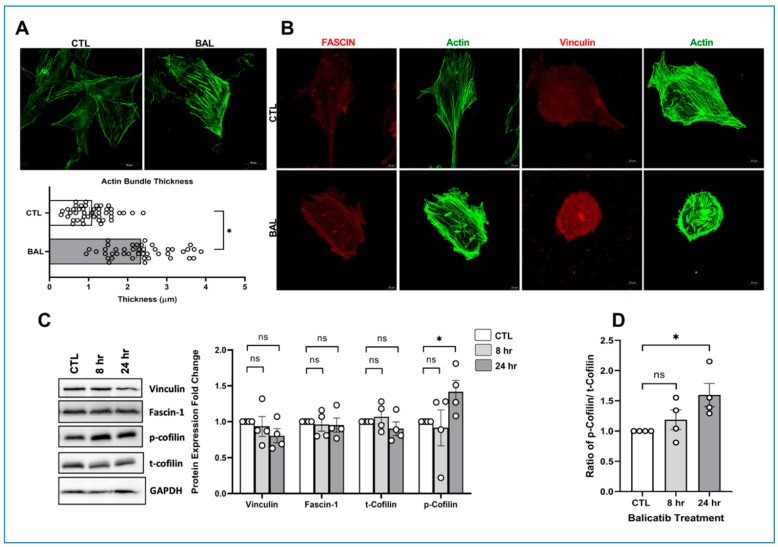
Effect of CTSK inhibition by balicatib on actin polymerization in PTM cells. (**A**) Analysis of actin cytoskeleton in response to inhibition of CTSK activity by 10 μM balicatib treatment for 24 h in serum-starved PTM cells. F-actin labeled with Alexa Fluor 488 phalloidin (green) in PTM cells treated with 10 μM balicatib demonstrates strong actin stress fibers with increased thickness, potentially due to actin-bundling. The bottom panel shows the graphical representation of the measured actin thickness (in μm) using ImageJ software. There is a significant increase in actin thickness in the balicatib treated cells compared to vehicle DMSO. (**B**) Distribution analysis of actin-bundling protein fascin and focal adhesion vinculin in response to inhibition of CTSK activity by balicatib. Immunofluorescence imaging showed a marked redistribution of fascin aligning on to actin stress fibers, and vinculin at the edges of the actin stress fibers in the balicatib treated PTM cells compared to the DMSO control. (**C**) Analysis of protein expression changes in fascin, vinculin, and cofilin in PTM cells treated with 10 μM balicatib treatment for 24 h. Balicatib treatment showed a significant increase in p-cofilin compared to control with no changes in vinculin, fascin-1, and t-cofilin. (**D**) Ratio of p-cofilin/t-cofilin was derived from the immunoblot analysis and demonstrated a significant increase in the ratio for balicatib treatment compared to DMSO control. GAPDH was immunoblotted as the loading control. Circles in the histograms represent sample numbers. Values represent mean ± SEM, where *n* = 4 (biological replicates). * *p* ≤ 0.05 was considered significant and n.s. denotes non-significant.

In addition to ECM remodeling, the balance between actin polymerization and depolymerization regulates the contractile properties of TM, and that is essential for regulating AH outflow via TM [29,31,82,83]. External signals drive changes in cellular contractility and mechanical properties in TM through an actomyosin-based cytoskeletal network [31]. Increased actomyosin assembly and focal adhesion formation has been found to influence plasticity and fibrogenic activity in the TM, and such signals can lead to elevated IOP [84]. Sustained elevation of IOP can also induce cytoskeletal remodeling, which further increases outflow resistance [73]. To investigate if CTSK inhibition by balicatib brought about cytoskeletal changes, we first probed for actin using phalloidin labeling in PTM cells. As seen in Figure 4A (top panel), 24 h of 10 μM balicatib treatment on PTM cells demonstrated a strong qualitative increase in actin stress fibers (right panel) compared to vehicle DMSO treated (left panel). On quantitatively measuring the thickness of stress fibers using ImageJ software depicted in the graph, we identified that balicatib treatment-induced actin stress fiber thickness (*n* = 44, *p* = 0.0001) and actin-bundling significantly compared to vehicle DMSO (Figure 4A). Increased actin-bundle thickness is indicative of increased rigidity in the actin cytoskeleton [85,86].

Fascin is a 55 kDa protein involved in the formation of actin filament bundling and an important regulatory element in the maintenance and stability of parallel bundles of filamentous actin [87,88]. Since we found strong actin-bundling due to balicatib treatment, we investigated the distribution of fascin and focal adhesion protein vinculin in the presence and absence of balicatib. Immunofluorescence analysis showed that balicatib-induced fascin (red staining on the left bottom panel in Figure 4B) align with actin stress fibers (green labeling with phalloidin) and a marked reorganization of vinculin (red staining on the right panels in Figure 4B) at the edges of actin stress fibers in comparison with vehicle DMSO control (top panels in Figure 4B).

Cofilin is an important actin-binding protein that regulates actin filament dynamics by involving the severing and depolymerization of actin filaments [89]. Phosphorylation of cofilin makes it inactive and absence of its function induces stabilization of actin filaments and formation of actin stress fibers [90,91]. Since we found rearrangements in fascin distribution and actin-bundling, we evaluated the changes in the protein levels of vinculin, fascin, and the ratio of p-cofilin/t-cofilin using immunoblotting. The protein expression analysis (Figure 4C) did not show any change in expression of vinculin, fascin-1, and t-cofilin levels, but p-cofilin significantly increased (*n* = 4, *p* = 0.04) at 24 h of balicatib treatment. Additionally, there was a significant increase in the ratio of p-cofilin/t-cofilin in balicatib treatment for 24 h compared to the vehicle DMSO control (*n* = 4, *p* = 0.02) (Figure 4D). Therefore, these data on strong actin-bundling via fascin and an increase in the ratio of p-cofilin/t-cofilin under the loss of CTSK activity is indicative of increased cellular contraction with a reduction in depolymerization and stabilization of actin filaments. However, the question that we had here was if these changes in ECM and actin contractility were due to the modulation of CTSK activity or if it was a result of random off-target effects of balicatib.

### 3.4. Constitutive CTSK Expression Decreases ECM Levels, Their Distribution and Increases Actin Depolymerization in TM

**Figure 5:**
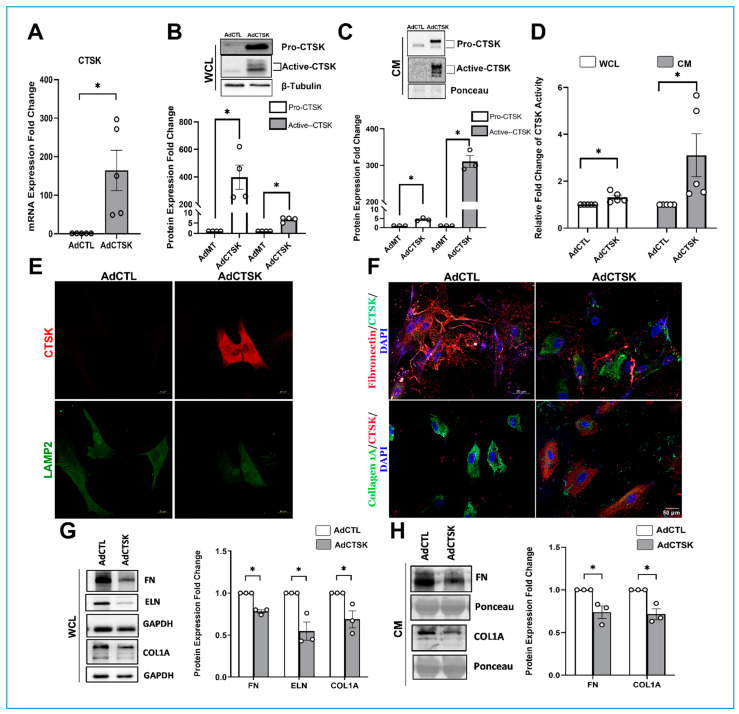
Effect of constitutive CTSK on the ECM expression and distribution in serum-starved HTM cells. (**A**) Quantification of changes in CTSK mRNA due to adenovirus-mediated constitutive expression of CTSK (AdCTSK) showing a significant increase in CTSK mRNA expression by ~164-fold compared to control (AdCTL). (**B**,**C**) Quantification of CTSK protein changes in WCL and CM in response to AdCTSK treatment. AdCTSK induced pro-CTSK and active-CTSK protein expression significantly in WCL and CM. Shown in the blot are two bands in the AdCTSK lanes upon resolving longer, indicating endogenous CTSK (bottom band) and the top band due to AdCTSK containing FLAG and His tag. Both endogenous and overexpressed CTSK bands from AdCTSK lanes were utilized. β-tubulin was used as a loading control for WCL, and Ponceau S-band at 55 kDa was used for the CM. (**D**) CTSK activity assay for changes in CTSK activity measured in WCL and CM from serum-starved HTM cells treated with AdCTSK and AdCTL. A significant increase in CTSK activity was seen in WCL and in CM. (**E**) Analysis of CTSK distribution in response to AdCTSK treatment in serum-starved HTM cells. Immunofluorescence imaging showed AdCTSK induced CTSK (Alexa Flour 568/red) (top panel) expression and distribution compared to AdCTL. No changes in lysosomal distribution were observed due to the increase in CTSK as identified by lysosome-associated membrane protein 2 (LAMP2) staining (Alexa Fluor 488/green) (bottom panel). Images were captured in z-stack in a confocal microscope, and stacks were orthogonally projected (scale bar = 20 microns). (**F**) Qualitative analysis of changes in ECM distribution in response to AdCTSK treatment in serum-starved HTM cells. Representative image—The top right panel shows a marked decrease in the FN distribution (red) upon an increase in CTSK (green) due to AdCTSK, and the bottom panel shows a marked decrease in collagen immunopositivity (green) upon an increase in CTSK (red) in comparison with panels on the left AdCTL. Images were captured in z-stack in a confocal microscope, and stacks were orthogonally projected. (**G**,**H**) Quantification of AdCTSK-mediated ECM protein changes in WCL and CM. Analysis of WCL showed a significant reduction in ECM protein expression of FN, elastin (ELN), and COL1A compared to AdCTL. In CM, a significant reduction in secreted FN and COL1A was observed in AdCTSK compared to AdCTL. The results were based on immunoblot analysis with subsequent densitometric analysis. For WCL, GAPDH was immunoblotted as the loading control. For secretory proteins in CM, the Ponceau S protein band (~55 kDa), which did not show variability across treatments, was considered as the loading control, and it represents equal loading. Circles in the histograms represent sample numbers. Values represent the mean ± SEM, where *n* = 3–5 (biological replicates). * *p* ≤ 0.05 was considered significant and n.s. denotes non-significant.

To understand the gain of a functional role of CTSK in the regulation of ECM remodeling and cytoskeletal organization, we utilized adenovirus mediated constitutive CTSK (AdCTSK) expression in TM. HTM cells were either treated with AdCTSK or control empty virus (AdCTL), which does not bring about any changes at the cellular level. Firstly, we established that AdCTSK increased CTSK mRNA expression significantly (*n* = 5, *p* = 0.01) (Figure 5A). AdCTSK increased the CTSK protein levels in WCL, pro-CTSK (*n* = 4, *p* = 0.004) and active-CTSK (*n* = 4, *p* = 0.0001) (Figure 5B), and secretory CTSK protein levels in CM, pro-CTSK (*n* = 3, *p* = 0.002) and active-CTSK (*n* = 3, *p* = 0.0001), compared to AdCTL (Figure 5C). WCL and CM from AdCTSK transduced HTM cells showed two bands upon resolving longer, indicating endogenous CTSK (bottom band) and the top band due to AdCTSK containing FLAG and His tag (additional ~3 kDa). For calculation purposes, both endogenous and overexpressed CTSK bands from AdCTSK lanes were utilized.

Therefore, we established that AdCTSK is increasing CTSK expression significantly. Further, we assessed the CTSK activity changes due to AdCTSK. Figure 5D shows that the relative fold change of CTSK activity in AdCTSK was increased significantly in WCL (*n* = 5, *p* = 0.01) and in CM (*n* = 5, *p* = 0.05). Further, immunofluorescence imaging for CTSK (in red) showed increased CTSK distribution in AdCTSK compared to AdCTL (top panel) (Figure 5E). Since CTSK is a lysosomal cysteine protease, we looked for changes in lysosomal distribution under AdCTSK using a lysosomal marker, lysosome-associated membrane protein 2 (LAMP2), and staining. Figure 5E shows the distribution of LAMP2 (green panel) in AdCTL (left panel) and AdCTSK (right panel). No prominent change was observed in the AdCTSK compared to AdCTL, indicating that a constitutive increase in CTSK did not alter the lysosomal distribution in TM.

CTSK is involved in ECM remodeling [41,92], and our results from balicatib-mediated blockage of CTSK activity resulted in increased ECM proteins. To understand the role of CTSK in ECM remodeling, we studied the effects of AdCTSK on the distribution and levels of ECM proteins. Under CTSK overexpression using AdCTSK in HTM cells, immunofluorescence labeling of ECM proteins (Figure 5F)—FN (in red) (top panel) and COL1A (in green) (bottom panel)—demonstrated a comprehensive decrease in immunolocalization and distribution. Thus, denoting the loss of intracellular and fibrillar ECM in AdCTSK compared to AdCTL. Further, the WCL protein expression analysis (Figure 5G) showed a significant reduction of FN (*n* = 3, *p* = 0.01), ELN (*n* = 3, *p* = 0.01), and COL1A (*n* = 3, *p* = 0.04) in AdCTSK compared to AdCTL. In addition, secretory ECM levels in CM (Figure 5H) demonstrated a significant decrease in FN (*n* = 3, *p* = 0.02) and COL1A (*n* = 3, *p* = 0.01) in AdCTSK treatment compared to AdCTL. These results indicate the CTSK gain of function can lower the intracellular and the secreted ECM levels, as well as their distribution. Thus, suggesting the important contribution of CTSK in regulating the ECM remodeling in the TM outflow pathway.

**Figure 6:**
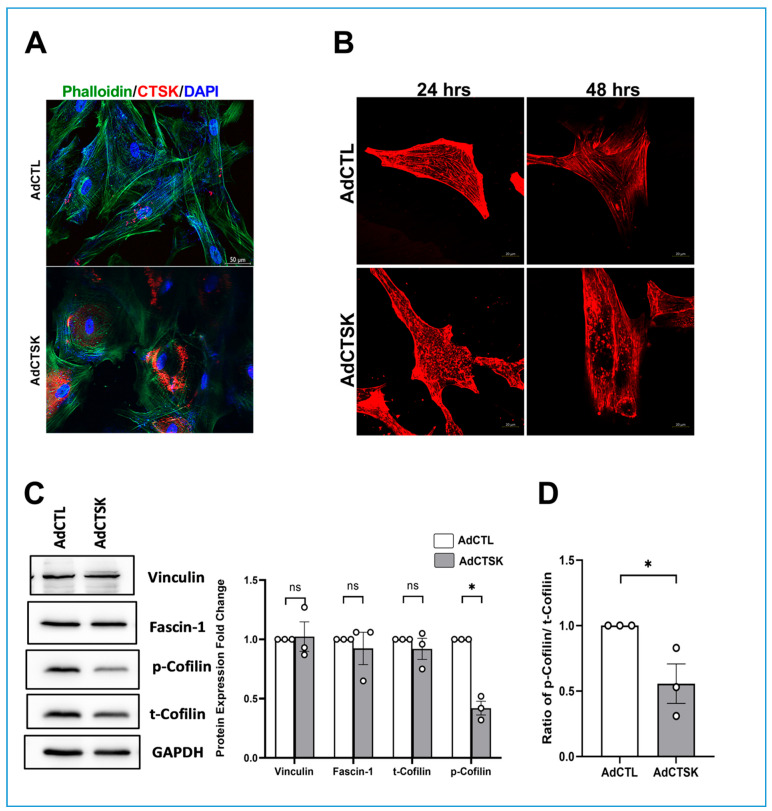
Effect of CTSK expression on actin polymerization in HTM cells. (**A**) Analysis of actin in response to AdCTSK in serum-starved HTM cells. Immunofluorescence imaging of CTSK showing AdCTSK-induced CTSK expression (Alexa Flour 568/red) (bottom panel) compared to AdCTL. F-actin labeled with phalloidin (green) in HTM cells treated with AdCTSK showed weak actin stress fiber staining with actin coalescing compared to AdCTL. Nuclear staining is shown by DAPI staining (blue). Images were captured in z-stack in a confocal microscope, and stacks were orthogonally projected. (**B**) SiR-actin based live-cell imaging to monitor changes in polymerized actin in response to AdCTSK. F-actin stained with SiR-actin demonstrated loss of actin fibers at 24 and 48 h after AdCTSK treatment (bottom panels) in HTM cells compared to AdCTL (top panels). Images were captured as a z-stack in a confocal microscope and the stacks were rendered using orthogonal projection. (**C**) Analysis of protein expression changes in fascin, vinculin, and cofilin in HTM cells treated with AdCTSK and AdCTL. AdCTSK treatment showed a significant reduction in p-cofilin compared to control with no changes in vinculin, fascin-1, and t-cofilin. (**D**) The ratio of p-cofilin/t-cofilin was derived from the immunoblot analysis and demonstrated a significant decrease in AdCTSK compared to AdCTL. The results were based on immunoblot analysis with subsequent densitometric analysis. GAPDH was immunoblotted as the loading control. Circles in the histograms represent sample numbers. Values represent mean ± SEM, where *n* = 3 (biological replicates). * *p* ≤ 0.05 was considered significant and n.s. denotes non-significant.

Next, to understand the significance of CTSK on actin cytoskeletal organization, we studied the effect of AdCTSK on actin polymerization using two different methods. Serum-starved HTM cells transduced with AdCTSK or AdCTL, cells were fixed 72 h later, and F-actin was labeled with phalloidin. In AdCTSK, actin (in green) (Figure 6A) (bottom panel) showed coalesced structures with the improper formation of filamentous actin compared to AdCTL, which showed clear F-actin fibers. To better visualize the changes in actin structures in real-time, F-actin was stained with SiR-actin, a fluorogenic, cell-permeable, and highly specific actin probe. Live-cell imaging was performed to record the changes in F-actin for 24 and 48 h of AdCTSK treatment in HTM cells and compared with AdCTL treatment. Both 24 and 48 h of AdCTSK treatment, Figure 6B (bottom panels), showed a reduction in actin stress fibers compared to AdCTL. Finally, we analyzed the changes in expression of actin-bundling protein fascin-1, focal adhesion protein vinculin, and t-cofilin and the p-cofilin due to AdCTSK. We found no changes in the levels of fascin, vinculin, and t-cofilin between AdCTL and AdCTSK (Figure 6C). Interestingly, p-cofilin levels were significantly decreased in AdCTSK compared to AdCTL (*n* = 3, *p* = 0.0006) (Figure 6D). The ratio of p-cofilin/t-cofilin was significantly reduced (*n* = 3, *p* = 0.04) (Figure 6E), indicating an increase in actin depolymerization. These findings confirmed that CTSK plays a direct role in regulating ECM homeostasis and actin-cytoskeletal organization in TM.

### 3.5. Constitutive CTSK Expression Regulates the TGFβ2 Bioavailability by Modulating the Presence of Active TGFβ2

**Figure 7:**
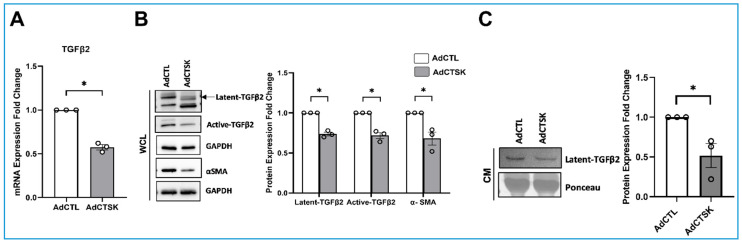
Effect of constitutive CTSK expression on TGFβ2 levels in HTM cells. (**A**) Analysis of TGFβ2 mRNA expression in response to AdCTSK in serum-starved HTM cells. TGFβ2 mRNA levels were significantly decreased in AdCTSK treatment compared to AdCTL. GAPDH was used as an internal control. (**B**) Analysis of TGFβ2 and αSMA protein expression in response to AdCTSK. A significant decrease in latent-TGFβ2 (top band), active-TGFβ2, and αSMA was observed in WCL. GAPDH was immunoblotted as the loading control. (**C**) AdCTSK treatment significantly reduced latent-TGFβ2 protein levels in CM. The ponceau S protein band that did not show variability across treatments (~55 kDa) was considered as a loading control, and it represents equal loading. Circles in the histograms represent sample numbers. Values represent the mean ± SEM, where *n* = 3 (biological replicates). * *p* ≤ 0.05 was considered significant and n.s. denotes non-significant.

TGFβ2 is a pro-fibrotic growth factor involved in the glaucoma pathogenesis, and it is found to be elevated in the AH of glaucoma patients [59,93]. TGFβ2 is also reported to elevate IOP [60,62]. Interestingly, CTSK is reported to regulate TGFβ and vice versa in other organisms and tissues [55]. We tested the effect of increased CTSK levels using AdCTSK expression on TGFβ2 levels. Serum-starved HTM cells treated with AdCTSK for 72 h were assessed for changes in TGFβ2 mRNA and protein levels. AdCTSK compared to AdCTL demonstrated a significant reduction in total TGFβ2 mRNA levels (Figure 7A) (*n* = 3, *p* = 0.0001). The protein expression analysis from WCL showed a significant reduction in the latent TGFβ2 (~50 kDa) (*n* = 3, *p* = 0.0003), and the active-TGFβ2 (~25 kDa) (*n* = 3, *p* = 0.001) (Figure 7B). In the CM (Figure 7C), we observed a significant reduction in latent-TGFβ2 (*n* = 3, *p* = 0.03). The CM showed very faint bands for active-TGFβ2, and densitometry was difficult to perform (data not shown). Studies show that TM cells are highly contractile and express a baseline αSMA level. By the process of endothelial mesenchymal transition (EndMT), upon activation by TGFβ2, TM cells have the propensity to transdifferentiate into αSMA-, fibroblast specific protein (FSP1)-, and collagen-1-expressing myofibroblast-like cells indicating a fibrotic response [31,94]. The inhibition of TGFβ2 signaling can attenuate the EndMT process in TM cells [94]. We found that HTM cells treated with AdCTSK showed a significant reduction in αSMA expression (*n* = 3, *p* = 0.01) compared to AdCTL (Figure 7C). These results demonstrate that CTSK possesses an antifibrotic effect in TM by regulating TGFβ2 and αSMA expression.

## 4. Discussion

This proof-of-concept study demonstrates that a direct perturbation of the activity or function of the ECM degrading secretory lysosomal enzyme CTSK can elevate IOP by altering the ECM degradation. To our knowledge, this is the first study to establish the role of CTSK as an important regulator of actin cytoskeleton-based contractile force and ECM stiffness in the outflow pathway to aid in the IOP homeostasis. We first report the expression and distribution of CTSK in the human AH outflow pathway and its secretion into the AH. The pharmacological inhibition of CTSK activity increased the ECM levels in TM and increased the IOP. We show that CTSK activity inhibition and overexpression, along with the availability of active CTSK, induced actin polymerization and depolymerization, respectively, by changing the phosphorylation of cofilin at serine 3. Our results reinforce the significance and the role of actin cytoskeleton—ECM interactions in the regulation of IOP homeostasis. Finally, the increase in total CTSK and the presence of active CTSK can negatively regulate the availability of functional TGFβ2, indicating a potential antifibrotic function of CTSK. Collectively, we propose that the activation of CTSK to release the actin cytoskeletal tension and lower ECM stiffness is an attractive therapeutic strategy to lower IOP.

The major changes in glaucomatous eyes are excessive alterations in ECM organization in the JCT-TM region and the accumulation of sheath-like plaque materials lead to altered stiffness [13,14,16]. An optimal balance in ECM remodeling in the JCT-TM region is needed for the generation of AH resistance, but excess ECM deposition results in increased resistance and elevated IOP. Histological analyses of human autopsy glaucoma eyes have provided evidence for significantly increased COL1A and total collagen in the TM outflow pathway in glaucomatous eyes compared with the age-matched controls [13]. Thus, implying an association between the activation of fibrogenic activity in the AH outflow pathway and POAG. Therefore, studying the role of the potent collagenase CTSK [95] in AH outflow is very important. Our data on CTSK expression and distribution in cultured TM cells, human TM tissue, and the secreted CTSK in AH confirms the expression, distribution, secretion, and presence of pro- and active-CTSK. Further, establishing the fact that pro-CTSK is secreted in an inactive form and must be activated to be functional in the extracellular milieu. Further studies are required to clarify if activated CTSK is secreted from the TM cells and/or if secretory lysosomes from TM cells are stimulated to fuse to the membrane and release their contents [96] in AH, based on the need. We found that CTSK expression is tightly regulated by mechanical stress, as well as in response to ocular hypertension inducing factors, including DEX, TGFβ2, Endo-1, and CTGF. We found that these stressors, except elevated IOP, which increased the active CTSK, lowered the CTSK expression. Thus, suggesting that these stressors are known to increase ECM negatively regulate CTSK expression to promote their fibrotic effects. Future studies are needed to determine the regulation, mechanism of the secretion, and CTSK activation paradigm by the IOP elevating fibrogenic factors.

Interestingly, CTSK activity has the unique ability to cleave both helical and telopeptide regions of type 1 collagen [42], fibronectin [97], and elastin [98]. Since we saw a significant decrease of CTSK levels by stressors, we hypothesized that the loss of CTSK function can induce ECM levels in the TM outflow pathway. Therefore, we utilized balicatib, a pharmacological inactivator of CTSK, to study the role of CTSK in ECM and IOP regulation. The treatment of balicatib on TM cells did not induce cell death, nor did balicatib compensate for the loss of CTSK activity by inducing cathepsin B since balicatib is a known lysosomotropic agent. In vitro studies showed that balicatib significantly increased both FN and COL1A by 24 h of CTSK activity inhibition. Additionally, ex vivo studies by perfusion of balicatib in porcine eyes increased IOP by two-fold in 8 h and sustained until 20 h. It is unclear why the IOP starts to drop back towards the baseline after 20 h of balicatib perfusion despite an appreciable increase in the COL1A deposited in the TM outflow pathway. Studies using the CTSK knockout mice [55] should shed more light on the regulation of IOP and outflow facility via alterations in ECM. Still intriguing was the elevation in IOP within 4 h of balicatib perfusion because the stoichiometric changes in ECM synthesis, secretion, and remodeling in TM are currently not available. Our findings reveal that balicatib perfusion in the porcine anterior segment cultures (ex vivo) and PTM cells (in vitro) induces ECM changes. The differences in changes in the tissue ECM and cell culture-based (in vitro) ECM in response to CTSK inhibition can be attributed to the complexities in the ECM makeup and their response to balicatib ex vivo versus the TM cells adhered to the substrate on the plastic tissue culture plates in vitro. Forces on cells and tissues applied from outside can be sensed by the cells via focal adhesion anchored to load-bearing bundled actin stress fibers [99]. There is strong ex vivo and in vivo evidence in support of the association of cytoskeletal integrity within the TM-JCT cells, AH outflow via the conventional outflow pathway, and the IOP [10,29,82]. Evidence supporting this is based on the use of cytoskeleton modulating agents, such as actin-depolymerizing agents, inhibitors of myosin light-chain kinase, myosin II, protein kinase C, Rho GTPase, and the FDA approved Rho kinase inhibitors to lower IOP [29,82]. Therefore, our study investigated if balicatib mediated increase in IOP is associated with changes in actin polymerization and stability and focal adhesion organization within the cells. Taking an in vitro approach, we found that balicatib strongly induced actin-bundling with the reorganization of actin-bundling protein fascin and focal adhesion protein vinculin. Further, semi-quantitative immunoblotting identified a significant increase in the ratio of p-cofilin to t-cofilin. The increase in phosphorylation of cofilin on serine-3 indicates the inactivation of cofilin, leading to actin polymerization and stabilization of actin filaments, as well as the suppression of actin turnover [100]. Interestingly, the airway epithelium of mice lacking CTSK showed a significant increase in the expression of α-actin and vimentin, indicating increased cellular contraction [55]. These data from our studies here and the literature strongly suggest that the functional loss of CTSK activity by balicatib induces a feed-forward p-cofilin-actin polymerization-fascin-mediated cytoskeletal contractile activity along with increased ECM driving IOP elevation.

Since the inhibition of CTSK activity resulted in increased actin polymerization, focal adhesion recruitment, and ECM production and distribution in the TM outflow pathway, we hypothesized that the increase in CTSK expression would aid in actin depolymerization as well as ECM degradation. The constitutive expression of CTSK was achieved using AdCTSK. AdCTSK increased the levels of total and active CTSK in cell lysate and in the media, as well as the activity of CTSK. AdCTSK expression aided in a significant loss of ECM, including FN, ELN, and COL1A, which are important components of TM ECM. Actin analysis using phalloidin revealed a coalesced appearance after 72 h of AdCTSK expression, and further live-cell imaging analysis of actin using SiR-actin labeling showed a significant loss in the actin fibers by 24 and 48 h post AdCTSK transduction. We believe that the maintenance of the actin in a depolymerized state by AdCTSK is aided by the decreased p-cofilin levels. Among the various pathogenic factors responsible for POAG, TGFβ2 is elevated in AH of patients with POAG [59]. TGFβ2 primarily contributes to the structural changes in the ECM of the TM and optic nerve head, as characteristically seen in POAG [101]. The abnormal signaling by TGFβ represents the key early event in inducing the transition of a proportion of TM or SC cells into a matrix and αSMA producing myofibroblast-like contractile cells [84,94,102,103]. Therefore, modulating TGFβ2 availability in TM can be a potential ocular hypotensive strategy. It has been previously shown that in lung tissue, CTSK protects against matrix deposition during fibrosis [50]. Interestingly, the levels of CTSK expression are inversely related to the levels of TGFβ expression [55]. More recent reports suggest that increased CTSK is associated with abnormally high levels of TGFβ signaling, and CTSK acts upstream of TGFβ [94]. These reports suggest a link between TGFβ and CTSK. Investigating the effects of AdCTSK on TGFβ2, we found that both latent TGFβ2 and the active TGFβ2 decreased significantly in the WCL and the CM. We propose that this decrease resulted in a significant decrease in α-SMA, a fibrotic and EndMT marker [104,105]. Thus, furnishing the evidence that increasing the CTSK expression and activation provides a novel therapeutic option by modulating TGFβ2 signaling, regulating ECM homeostasis in the TM outflow pathway, and lowering IOP.

### Limitations of This Study

This study has used one CTSK inhibitor, balicatib, to inhibit the activity of CTSK from providing evidence for the role of CTSK in the TM outflow pathway. The use of other CTSK activity inhibitors will be able to further substantiate the data presented here. The utilization of siRNA in vitro can provide evidence for the functional knockdown of CTSK in addition to CTSK activity inhibition. Additionally, the use of whole-body CTSK knockout mice or conditional knockout mice can be a potential strategy to further study the effects of CTSK in vivo. Determination of changes in the AH outflow facility in addition to IOP changes will provide important information into the mechanistic evidence for IOP regulation by CTSK. Assessment of the changes in the integrin adhesome network due to CTSK inhibition or activation can aid in answering how the actin, focal adhesions, and ECM changes are brought about by CTSK. Thus, providing a novel molecular mechanism for CTSK in regulating TM actin-ECM dynamics.

## 5. Conclusions

Collectively, based on the observations provided in our study here, it is reasonable to conclude that CTSK has an important role in regulating IOP by modulating the ECM in the TM outflow pathway and the availability of active TGFβ2. Further studies are underway using CTSK knockout mice in combination with in vitro SMAD reporter assays and SMAD phosphorylation studies in TM. These studies will help us to decipher the role of CTSK in regulating the TGFβ2 signaling and attenuating the fibrogenic mechanisms in TM. Additional analysis of the levels of CTSK protein and its activity status in glaucomatous TM tissue samples and AH in comparison to control will help better understand the disease pathogenesis. Finally, we have identified a potent collagenase, CTSK, as an ideal target to increase the breakdown of ECM, such as COL1A, FN, and ELN, in the conventional AH outflow pathway. This can contribute to significant lowering in IOP and prevention of glaucoma. Currently, there are no direct pharmacological activators of CTSK available. Further identification of novel small molecule CTSK activators as therapeutic leads using high throughput screening will aid in activating CTSK that can be used in lowering IOP.

## Figures and Tables

**Table 1:** Oligonucleotide primers used in the q-PCR based transcript expression analysis.

Primer Name	Gene Name	Sequence (5′–3′)	NCBI Reference Sequence
hTGFβ2 F	Human Transforming Growth Factor β2	TTGACGTCTCAGCAATGGAG	NM_001135599.4
hTGFβ2 R		TTCGCCTTCTGCTCTTGTTT	
hCTSK F	Human Cathepsin K	CAGTGAAGAGGTGGTTCAGA	NM_000396.4
hCTSK R		AGAGTCTGGGGCTCTACCTT	
hGAPDH F	Human Glyceraldehyde 3-phosphate dehydrogenase	TGCACCACCAACTGCTTAGC	NM_002046.7
hGAPDH R		GGCATGGACTGTGGTCATGAG	
pCTSK F	Porcine Cathepsin K	GCAGAACCCCAGACTCTATTG	NM_214302.1
pCTSK R		TCAGACACACAATCCACCAG	
pHMBS F	Porcine Hydroxymethylbilane Synthase	AGGATGGGCAACTCTACCTG	NM_001097412.1
pHMBS R		GATGGTGGCCTGCATAGTCT	

## Data Availability

The data for all the experiments, results, and data presented in this manuscript is stored on a common drive provided by IUSM and will be available upon request.
